# Unprecedented Combination of Rare Degenerative Pathologies in an Octogenarian Ex‐Football Player

**DOI:** 10.1111/neup.70004

**Published:** 2025-03-13

**Authors:** Shelley L. Forrest, Nusrat Sadia, Mozhgan Khodadadi, Charles Tator, Robin Green, Maria Carmela Tartaglia, Gabor G. Kovacs

**Affiliations:** ^1^ Tanz Centre for Research in Neurodegenerative Disease University of Toronto Toronto Canada; ^2^ Laboratory Medicine Program & Krembil Brain Institute University Health Network Toronto Canada; ^3^ Canadian Concussion Centre, Krembil Brain Institute, University Health Network University of Toronto Toronto Canada; ^4^ Division of Neurosurgery, Toronto Western Hospital University of Toronto Toronto Canada; ^5^ KITE‐Toronto Rehab University Centre Toronto Canada; ^6^ University Health Network Memory Clinic & Krembil Brain Institute University Health Network Toronto Canada; ^7^ Edmond J. Safra Program in Parkinson's Disease, Rossy Program for PSP Research and the Morton and Gloria Shulman Movement Disorders Clinic, Toronto Western Hospital Toronto Canada; ^8^ Department of Laboratory Medicine and Pathobiology and Department of Medicine University of Toronto Toronto Canada

**Keywords:** chronic traumatic encephalopathy, concussion, globular glial tauopathy, mixed pathology

## Abstract

A 79‐year‐old former professional football player presented with language deficits and cognitive changes. A year later, he had difficulty completing sentences, and 3 years after onset, was reduced to one‐word answers. He developed severe apathy and agitation, and became more impulsive. He eventually became mute and had difficulty with walking and balance. The patient had mild repetitive head injury while playing football and three concussions. Magnetic resonance imaging revealed left > right frontotemporal atrophy. Duration of illness was 6 years. Neuropathology revealed an unexpected number and diversity of degenerative pathologies, including chronic traumatic encephalopathy (CTE, high level), high level Alzheimer's disease neuropathologic change (A3B3C3), limbic Lewy body disease, cerebral amyloid angiopathy (type 2), argyrophilic grain disease (Stage 2), and neuronal intranuclear hyaline inclusion body disease. In addition, there was selective and asymmetric involvement of the corticospinal tract with globular oligodendroglial tau pathology corresponding to globular glial tauopathy (Type II). The patchy and irregular accentuation of cortical tau pathology, particularly in the depths of sulci and accumulation around blood vessels, allows the diagnosis of CTE‐neuropathologic change. This diagnosis correlated with the past medical history of multiple concussions. In addition, the patient had an unprecedented number and combination of additional degenerative pathologies, including those that are rare, and how they contributed to the clinical symptoms is difficult to interpret. Globular glial tauopathy Type II is a rare disorder that has been mostly reported in association with progressive supranuclear gaze palsy, and these observations support the notion that globular glial tauopathy Type II is an independent entity with isolated corticospinal tract involvement. These observations highlight that rare disorders can occur in the same individual and be overlooked, especially when there is more obvious pathology. It is essential for neuropathologists to consider an extensive array of neuropathological examinations when assessing patients with neurodegenerative disorders.

**Abbreviations**
AD: Alzheimer's disease
AGD: argyrophilic grain disease
CTE‐NC: chronic traumatic encephalopathy‐neuropathologic change
G‐B: Gallyas‐Braak
GGT: globular glial tauopathy
LATE‐NC: limbic predominant age‐related TDP‐43 encephalopathy‐neuropathologic change
LFB‐H&E: Luxol fast blue‐hematoxylin and eosin
MOCA: montreal cognitive assessment
NIHIBD: neuronal intranuclear hyaline inclusion body disease

## 1 Introduction

Chronic traumatic encephalopathy (CTE) is a neurodegenerative disorder predominantly described in professional athletes and military personnel following mild repetitive head injury. The pathognomonic lesion consists of phosphorylated (p)‐tau aggregates in neurons, astrocytes, and cell processes around small vessels in an irregular pattern at the depths of the cortical sulci [1]. A second consensus study made refinements to the description, emphasizing that p‐tau aggregates in neurons, with or without thorn‐shaped astrocytes, at the depth of a cortical sulcus are to be considered as CTE‐neuropathologic change (NC) [2]. Four stages have been proposed based on the severity and extent of pathological lesions [3]. Concomitant TDP‐43, amyloid‐β, α‐synuclein, and vascular pathologies are common in neurodegenerative and community‐based longitudinal aging cohorts [4]. The presence of mixed pathologies is associated with cognitive impairment and contributes to diverse and complex clinical symptoms. In aging and in CTE, the most frequent co‐pathologies include Lewy body disease, Alzheimer's disease (AD)‐related neuropathologic change, and TDP‐43 pathology predominantly in the limbic system called limbic predominant age‐related TDP‐43 encephalopathy (LATE‐NC). However, rare combinations of co‐pathologies, for example, with neuronal intranuclear hyaline inclusion body disease (NIHIBD) are also reported [5]. We report an ex‐football player with primary progressive aphasia and an unprecedented combination of degenerative pathologies, including CTE‐neuropathological change. This case was included in a recent patient series [6], however, the details of the neuropathology have not been described.

## 2 Clinical Summary

**FIGURE 1:**
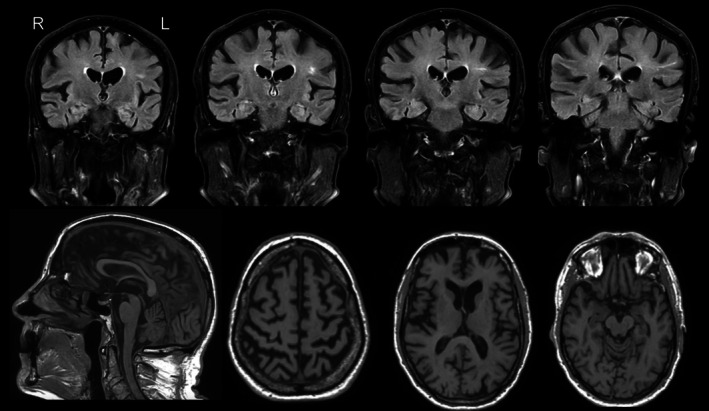
Representative MRI images taken when the patient was aged 81 years. MRI revealed mild asymmetric left greater than right frontotemporal atrophy and some linear white matter hyperintensity in the left frontal area.

An 85‐year‐old male ex‐football player who played 4 years in the Canadian Football League had pledged his brain to the Canadian Concussion Centre and, upon death, was examined. A post‐mortem interview with the next‐of‐kin revealed that the patient had developed language impairment at the age of 75 years with initial word‐finding difficulties with some mild mispronunciation, and slurring of speech. He also developed some difficulty with reading and writing. Three years after onset, he developed mild irritability, and cognitive assessment showed a Montreal Cognitive Assessment (MOCA) score of 21/30. He was diagnosed with logopenic variant primary progressive aphasia and started on an acetylcholinesterase inhibitor, donepezil. There was progression of symptoms over the next 5 years with increasing comprehension deficits, so he became dependent for instrumental activities of daily living. In the last 2 years before death, the patient became more impulsive, with some outbursts with family and friends, and developed severe apathy, agitation, and restlessness. At 79, 4 years after onset, he developed difficulty with walking and balance, had falls, and right sided weakness of extremities. He became mute and dependent for basic activities of daily living in the last 2 years before death. He had hallucinations and difficulty swallowing in the last year of life. The patient started to play contact sports including hockey in elementary school and had a history of three concussions: the concussion at age 11 and 21 included loss of consciousness, and the third, in his mid‐20s from a water ski accident was associated with post‐traumatic amnesia. He was also a victim of child abuse, but the specifics were not known to his next of kin. There was no known relevant family history of neurological disease. Magnetic resonance imaging at 81 years of age revealed mild frontotemporal atrophy in some gyri more on the left side, and some linear white matter hyperintensity in the left frontal area (Figure 1). A CT scan at the age of 85 showed mild progression of atrophy.

## 3 Pathological Findings

Histopathological analysis was carried out using formalin‐fixed, paraffin‐embedded, 4.5‐μm‐thick sections from various regions of the brain, including the cerebral cortex and white matter (frontal, temporal, parietal, occipital), hippocampus, amygdala, basal ganglia (caudate nucleus, putamen, globus pallidus), thalamus, subthalamus, and the brainstem (midbrain, pons, medulla oblongata). Histological examination was performed using Luxol fast blue‐hematoxylin and eosin (LFB‐H&E) as well as Gallyas‐Braak (G‐B). Immunohistochemistry was performed using the following primary antibodies: p‐tau (clone AT8, pSer202/Thr205, 1:1000, Invitrogen/ThermoFisher, Carlsbad, USA); antibodies against 3R‐tau (RD3, clone 8B6/C11, 1:2000, MilliporeSigma); 4R‐tau (RD4, clone 1E1/A6, 1:200, MilliporeSigma); Aβ (Clone 6F/3D, 1:50, Dako/Agilent, Santa Clara, USA); α‐synuclein (clone 5G4, 1:4000, Analytikjena, Jena, Germany); and phosphorylated (p)‐TDP‐43 (clone 11–9, 1:2000, CosmoBio, Tokyo, Japan). Immunostaining was performed using the Dako Autostainer Link 48 and EnVision FLEX+ Visualization System, according to the manufacturer's instructions. Subsequently, all sections were counterstained with hematoxylin.

**FIGURE 2:**
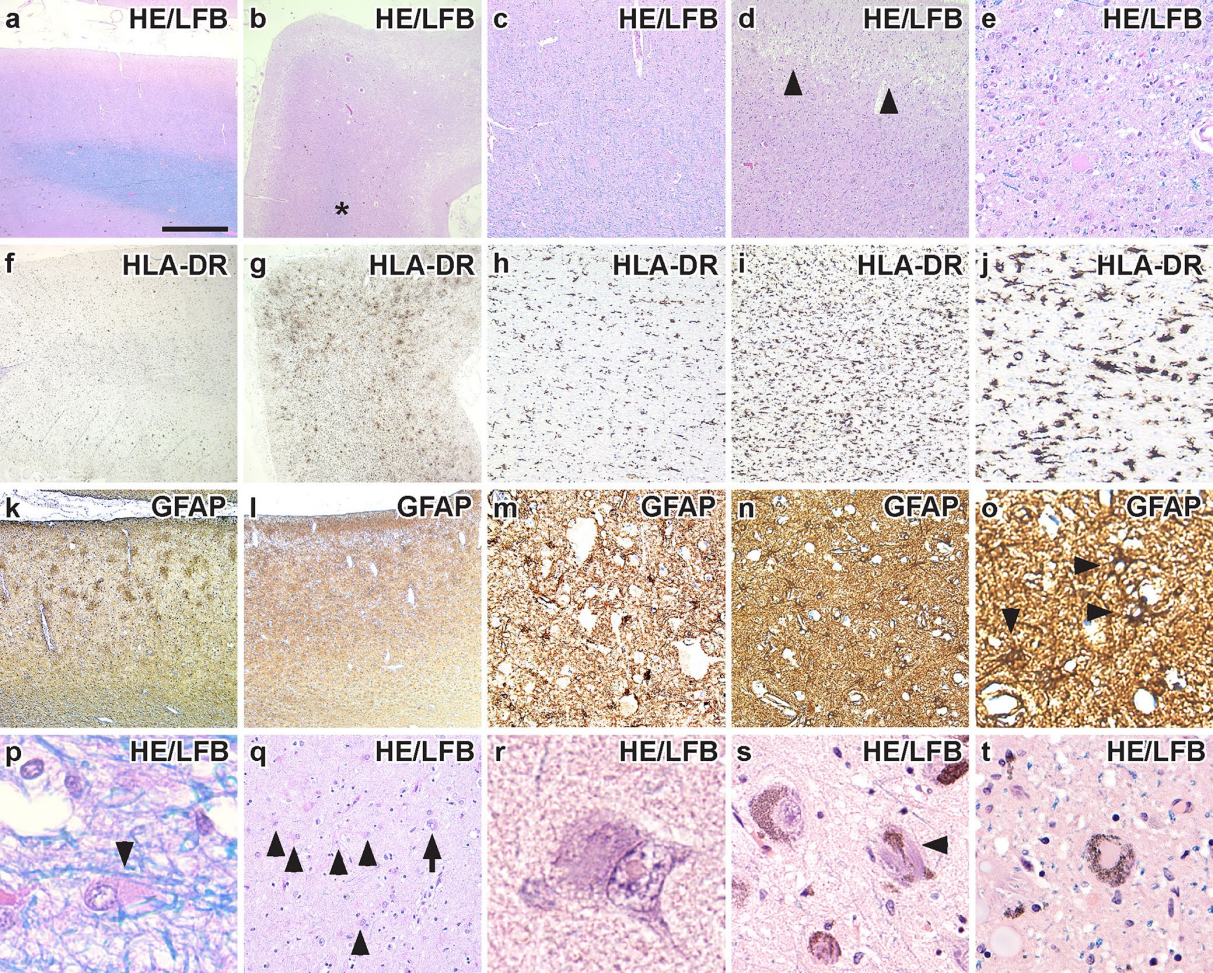
Cortical and subcortical pathological alterations. (a, b) Low magnification of the right (a) and left (b) motor cortex showing the asymmetric neuronal loss and white matter (*) demyelination. (c, d) Higher magnification of the right (c) and left (d) motor cortex. The left motor cortex had marked neuronal loss and superficial spongiosis (arrowheads). (e) Severe gliosis in the left motor cortex. (f, g) Low magnification of the right (f) and left (g) motor cortex immunostained with HLA‐DR. The left motor cortex had massive microglial upregulation. (h‐j) Higher magnification comparing HLA‐DR‐immunostaining in the right (h) and left (i, j) motor cortex. (k, l) Comparison of GFAP‐immunostaining in the right (k) and left (l) motor cortex. (m‐o) Higher magnification comparing GFAP‐immunostaining in the right (m) and left (n, o) motor cortex. The left motor cortex has severe GFAP‐immunostaining. (p) Oligodendroglia with swollen eosinophilic cytoplasm were observed in the white matter underlying the left motor cortex. (q) The hippocampus was gliotic, shown here are reactive astrocytes (arrowheads) in the hippocampal CA1 subregion. Neurofibrillary tangles (arrow) were also observed in this region. (r) Round eosinophilic intra‐nuclear inclusion in a neuron in the hippocampal CA4 subregion. (s) Neurofibrillary tangles (arrowhead) were observed in pigmented neurons in the substantia nigra. (t) Pale bodies were also observed in the locus coeruleus. Scale bar in a represents 500 μm and also applies to b, f, g, k and l; 250 μm in c, d, h and i; 100 μm in e, j, m, n and q; 40 μm in o and t; 15 μm in p; 8 μm in r; 50 μm in s.

Detailed neuropathological examination revealed that neurodegeneration was most severe in the frontal and temporal cortices. The left primary motor cortex had severe neuronal loss, gliosis, and superficial spongiosis (Figure 2a–e), which was accompanied by massive HLA‐DR (Figure 2f–j) and GFAP (Figure 2k–o) immunoreactivity. Oligodendroglia with swollen, eosinophilic cytoplasm were also observed in the white matter underlying the left motor cortex (Figure 2p). In contrast, the right motor cortex was well‐preserved with normal cytoarchitecture intact. The hippocampus had normal morphology. The hippocampal CA1 region contained reactive astrocytes and neurofibrillary tangles (Figure 2q), and round eosinophilic intranuclear inclusions were observed in the CA1 and CA4 regions (Figure 2r). The basal ganglia and thalamus had regular neuronal morphology and a lack of reactive astrogliosis. There was mild neuronal loss in the substantia nigra and locus coeruleus with neurofibrillary tangles (Figure 2s) and pale bodies (Figure 2t) present. The cerebellar cortex showed patchy Purkinje cell loss, and the dentate nucleus was unremarkable.

**FIGURE 3:**
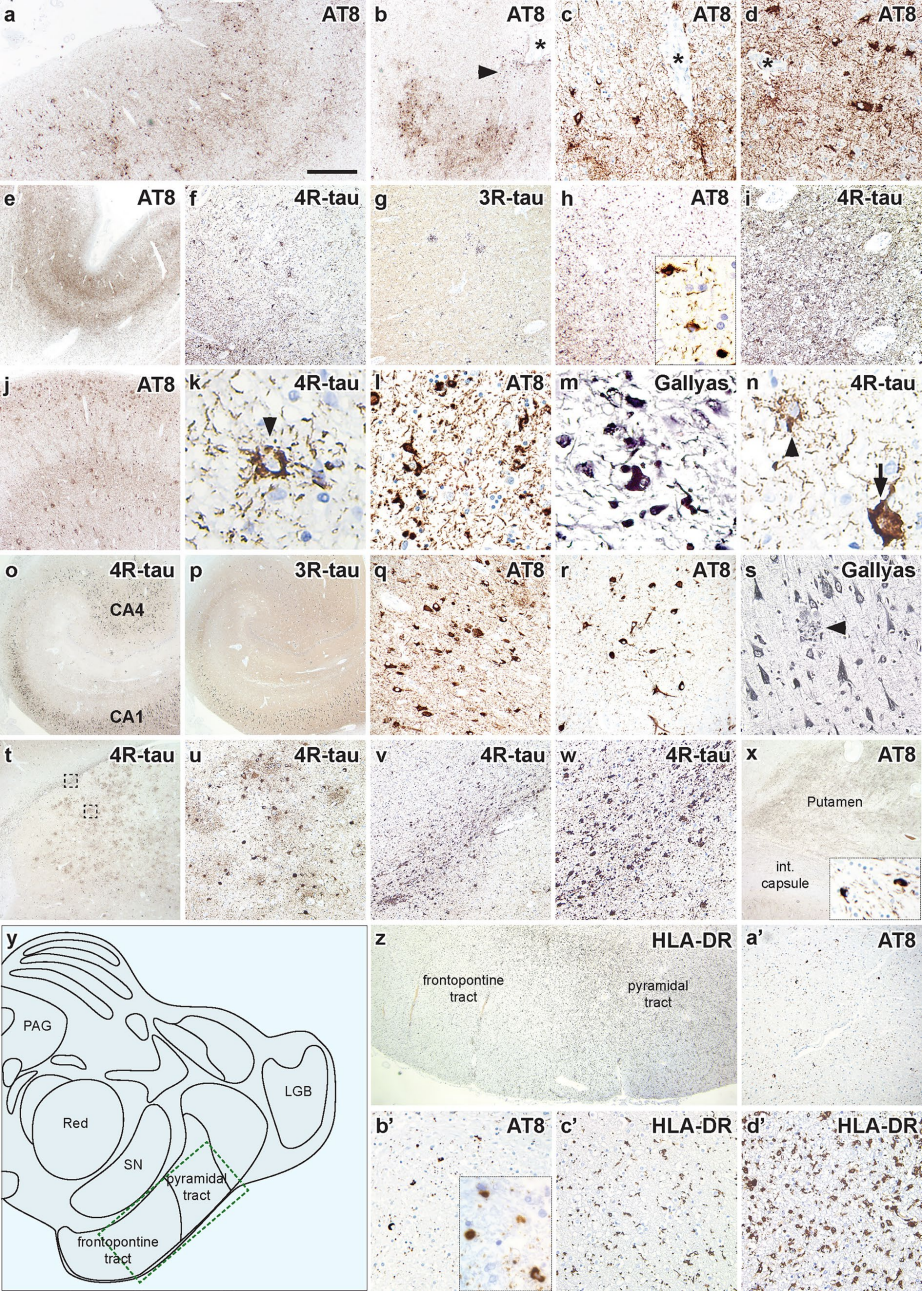
Distribution and extent of chronic traumatic encephalopathy and globular glial tauopathy Type II pathological features. (a, e, j) Severe tau pathology was observed in all cortical regions examined, including the frontal (a), motor (e) and temporal (j) cortices. (b) The depths of cortical sulci contained subpial (arrowhead) and gray matter astrocytic tau‐immunoreactivity and neurofibrillary tangles in patchy and irregular patterns, which clustered around perivascular regions. Sulcal groove indicated (*). (c, d) Perivascular accentuation of astrocytic‐immunoreactivity and neurofibrillary tangles around perivascular regions (*). (f) The left motor cortex had globular horse‐shoe shaped neuronal inclusions, globular astrocytic and oligodendroglial inclusions immunostained for 4‐repeat tau. (g) 3‐repeat‐immunostaining was absent. (h, i) Severe tau‐ and 4‐repeat immunostaining in the white matter underlying the motor cortex. (k‐n) Tau pathology in the motor cortex was characterized by neurons with globose and horseshoe (arrow in n) morphology, globular astrocytic (arrowheads in k and n) and oligodendroglial inclusions, which were immunopositive with 4‐repeat tau (k, n) and observed with Gallyas silver (m). l and m show white matter pathology. (o, p) All hippocampal subregions had neurofibrillary tangles and neurites that were immunoreactive for 3‐repeat (o) and 4‐repeat (p) tau. (q, r) Phosphorylated tau‐immunopositive neurons and neurofibrillary tangles in the hippocampal CA1 (q) and CA4 (r) subregions. Tau‐immunopositive neurons in the CA4 subregion contained dendritic swellings. (s) Gallyas silver staining revealed neurofibrillary tangles and neuritic plaques (arrowhead) in the hippocampus. (t) The amygdala contained patchy 4‐repeat tau‐immunostained neurons, threads and astrocytes. Dashed boxes in (t) are enlarged in (u) and (v) showing the gray matter and white matter thorn‐shaped astrocytes, respectively. (w) Higher magnification of the thorn‐shaped astrocytes in white matter age‐related tau astrogliopathy. (x) Phosphorylated tau pathology in the putamen and internal capsule. Inset shows a higher magnification of globular oligodendroglial inclusions in the internal capsule. (y) Diagram of the midbrain showing the location of the frontopontine and pyramidal tracts. (z’) There was a higher density of HLA‐DR immunoreactivity in the pyramidal tract compared to that observed in the frontopontine tract. (a’, b’) Low (a’) and higher (b’) magnification images showing AT8‐immunostained globular glial inclusions in the pyramidal tract. (c’, d’) Comparison of HLA‐DR‐immunostaining in the frontopontine (c’) pyramidal (d’) tracts. A higher density was observed in the pyramidal tract. Scale bar in a represents 250 μm and also applies to b, t and x; 60 μm in c and d; 100 μm in k, 50 μm in l and m, 75 μm in n; 500 μm in e, j, o and p; 150 μm in f‐i, q‐s, u and v; 125 μm in w; 200 μm in z; 150 μm in a’; 70 μm in b’‐d’.

Severe tau pathology including threads, neurites, and neurofibrillary tangles, was observed in all cortical regions examined, including superficial layers (Figure 3a,e,j). The primary visual cortex was mildly affected. In addition, the depths of cortical sulci contained subpial and gray matter astrocytic tau‐immunoreactivity and neurofibrillary tangles in patchy and irregular patterns, which clustered around perivascular regions (Figure 3b–d). Clusters of thorny astrocytes were observed in lobar white matter. The left motor cortex had tau‐immunopositive globular horseshoe shaped neuronal inclusions, globular astrocytic, and severe oligodendroglial inclusions (Figure 3f–i,k–n), which were 4‐repeat tau‐immunopositive (Figure 3f,i) and negative for 3‐repeat tau (Figure 3g). All hippocampal subregions contained neurofibrillary tangles and neurites that were immunoreactive for 3‐repeat and 4‐repeat tau, including the dentate gyrus (Figure 3o–r). These were also observed with G‐B silver (Figure 3s). Tangles in the CA4 subregion contained dendritic swellings (Figure 3r). The amygdala contained neurofibrillary and globose inclusions, threads, gray and white matter astrocytes, and oligodendroglial coiled bodies (Figure 3t–w). Globular oligodendroglial inclusions were seen in the internal capsule (Figure 3x). Neurofibrillary tangles were absent in the basal ganglia and subthalamic nucleus, although there was prominent tangle pathology in the midbrain tegmentum and substantia nigra. One side of the cerebral peduncles showed oligodendroglial tau inclusions, which were predominantly observed in the pontine descending tracts (Figure 3y,a’,b’). Immunostaining with HLA‐DR revealed severe microglial activation in the pyramidal tract compared with the frontopontine tract (Figure 3z,c’,d’), which corresponded to the higher density of globular oligodendroglial inclusions in the pyramidal tract. There was an absence of tau pathology in the cerebellar white matter and dentate nucleus.

**FIGURE 4:**
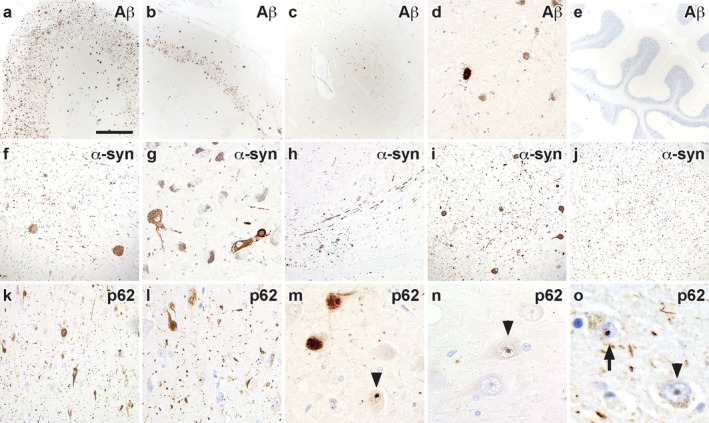
Additional immunohistochemical findings. (a‐e) Amyloid‐β plaques were present in the frontal cortex (a), hippocampus (b), striatum (c) and substantia nigra (d), and were absent in the cerebellum (e), corresponding to Thal phase IV. (f‐j) α‐synuclein‐immunopositive neurites and Lewy bodies were observed in the locus coeruleus (f), substantia nigra (g), the nerve root (h) of the dorsal motor nucleus of the vagus nerve (i), and the amygdala (j). (k‐o) p62‐immunopositive neurons and grains in the hippocampal CA1 region (k, l) and neuronal intranuclear inclusions (arrowheads) in the CA4 region (m, n). p62‐immunopositive astrocytic (arrow) and neuronal (arrowhead) intranuclear inclusions in the amygdala (o). P62‐immunopositive inclusions were absent in the cerebellum. Scale bar in a represents 500 μm and also applies to b and e; 250 μm in c and f; 100 μm in d and g; 125 μm in h‐j; 75 μm in k, 50 μm in l and m; 35 μm in n; 20 μm in o.

Amyloid‐β plaques were observed in cortical regions, the hippocampus, striatum, and substantia nigra, but were absent in the cerebellum (Figure 4a–e). Cerebral amyloid angiopathy was also present. α‐synuclein‐immunopositive neuronal inclusions and neurites were observed in the substantia nigra, locus coeruleus, dorsal motor nucleus of the vagus nerve and amygdala (Figure 4f–j). The hippocampus and amygdala contained tau‐ and p62‐immunopositive dendritic grains (Figure 4k,l), and p62‐immunopositive neuronal and astrocytic intranuclear inclusions were observed in the frontal cortex and medial temporal lobe, mostly in the hippocampal CA4 region (Figure 4m,n) and amygdala (Figure 4o), which were absent in the cerebellum. Immunostaining with phosphorylated‐TDP‐43 was negative in all regions.

## 4 Discussion

The patchy and irregular accentuation of cortical tau pathology, particularly in the depths of sulci and accumulation around blood vessels, allows the diagnosis of CTE‐NC with severity assessed as high CTE [3]. This diagnosis correlated with the past medical history of repetitive head injury. In addition, the patient had an unprecedented combination of additional pathologies, including those that are rare, that likely contributed to the complex clinical spectrum. High level Alzheimer's disease pathological change (A3B3C3) consisting of large amounts of amyloid‐β plaques in the neocortex, hippocampus, basal ganglia, and brainstem (Thal Phase 4), and neurofibrillary tangles corresponding to Braak stage V was found. Mild amyloid angiopathy (Type 2) was also present. AD‐neuropathologic change and cerebral amyloid angiopathy are commonly observed as mixed pathologies in community‐based, longitudinal aging studies and in major neurodegenerative diseases [4].

A further alteration is the neuron predominant alpha‐synucleinopathy in the form of Lewy body disease. This is characterized by neurites and neuronal pathological inclusions throughout the brainstem and amygdala, with an absence of Lewy bodies in neocortical areas. This corresponds to Stage 4 of Lewy related pathology according to Braak et al. [7] and limbic type of Lewy body disease using the recent Lewy pathology consensus evaluation criteria [8]. In addition, dendritic tau positive grains were observed in the hippocampus and amygdala with pretangles and oligodendroglial coiled bodies in the hippocampal white matter, which is compatible with argyrophilic grain disease (AGD, Saito Stage II) [9]. AGD is a common, predominantly age‐related tauopathy characterized by argyrophilic and tau‐immunopositive grains and oligodendroglial coiled bodies in the medial temporal lobe, which is thought to contribute to lowering the threshold for the development of cognitive decline [4]. AGD has also been reported to contribute to anxiety, depression, restlessness, and neuropsychiatric symptoms, potentially contributing to the complex clinical picture [10, 11].

A further peculiar tau pathology was recognized in the left motor cortex and descending corticospinal tract. Here, we detected a very selective and asymmetric involvement of the corticospinal tract with oligodendroglial tau pathology corresponding to globular glial tauopathy Type II. Globular glial tauopathies (GGT) were defined in a consensus study in 2013, and the three neuropathological subtypes (Types I–III) are characterized by tau‐immunopositive inclusions that are predominantly in oligodendroglia and/or astroglia in the frontal, temporal, and/or precentral cortices, with or without corticospinal tract involvement [12]. GGT Type II is a rare disorder [13] that has been associated with atypical progressive supranuclear palsy (PSP) and corticospinal tract degeneration [14]. However, we did not detect neuropathological features of PSP, and our observations here support the notion that GGT Type II is an independent entity from PSP with isolated, and in this case asymmetric, corticospinal tract involvement. Indeed, Tanaka et al. reported GGT‐type II cases from Japan, including peculiar neuronal morphologies that we also detected in our case, supporting this notion [15, 16]. Such pure involvement of the corticospinal system is very rare and even other subtypes of GGT (e.g., Type I) might co‐exist with Type II [17].

Finally, eosinophilic and p62‐immunopositive intranuclear inclusions in neuronal and astrocytic nuclei were detected in the frontal cortex and medial temporal lobe, corresponding to NIHIBD [18]. While the anatomical distribution of these inclusions is more restricted than reported in other cases [19], this is an unexpected finding and could be interpreted as incipient and it cannot be excluded that the repeated head trauma contributed to this unusual pattern. NIHIBD has young‐ and adult‐onset forms and is very rarely detected in the elderly [18]. In the adult form, a combination of dementia, parkinsonism, hyporeflexia, cerebellar signs, and autonomic dysfunction, and sphincter abnormalities are reported; however, the clinicopathological correlate of this relatively restricted and mild pathology, which most likely represents an early stage of the disease, is unclear in the present a case. Interestingly, NIHIBD has been reported in elderly individuals without clinical symptoms [20, 21], including in community‐based studies [20], or associated with rare conditions such as prion disease [5]. In younger individuals, trinucleotide GGC repeat expansions in the 5'UTR site of the *NOTCH2NLC* gene have been recognized as the pathogenesis of NIHIBD disease in Japanese and Chinese patients with juvenile and adult onset NIHIBD [22]. In addition to NIHIBD, GGG repeat expansions in *NOTCH2NLC* have been found in 12 of 101 Japanese patients with genetically unresolved adult leukoencephalopathy [23]. *NOTCH2NLC* repeat expansions have also been associated with Alzheimer's disease‐, frontotemporal dementia‐, and multiple system atrophy‐like phenotypes [24, 25].

Despite the prevalence of concomitant pathologies in longitudinal aging and neurodegenerative studies, the number and combination of degenerative pathologies in the current case, including rare forms, are exceptional, and how they contributed to the diverse clinical symptoms is difficult to interpret. These observations highlight that even rare disorders can occur in the same individual and can be overlooked when there is a more obvious pathology, for example, CTE and AD‐neuropathologic change. Importantly, the number of combined pathologies could not be suspected in MRI, although the asymmetric frontotemporal atrophy was suggestive of a neurodegenerative process. We propose that mild repetitive head injury induces p‐tau pathology and can accelerate diverse neurodegenerative proteinopathy forms in the same brain. It remains unclear whether repetitive head impacts from a young age and an initial concussion at age 11 contributed to an unusually high number of co‐pathologies in this case compared to those reported in other neurodegenerative diseases [26]. Current evidence suggests that a younger age of repetitive head impacts is associated with earlier cognitive and behavioral symptoms in former contact sports athletes but does not necessarily lead to more severe CTE [27]. However, it is still unknown whether earlier exposure increases the risk of developing a broader range of neurodegenerative pathologies.

## Ethics Statement

Approval of the research protocol: This study was approved by the UHN Research Ethics Board (Nr. 20‐5258) and University of Toronto (Nr. 39459) and was performed per the ethical standards established in the 1964 Declaration of Helsinki, updated in 2008.

## Consent

Brain tissue was obtained at autopsy through appropriate consenting procedures with informed consent from the patient or their relatives and approval of the local Ethical Committee.

## Conflicts of Interest

Gabor G. Kovacs is an Editorial Board member of Neuropathology Journal and a co‐author of this article. To minimize bias, they were excluded from all editorial decision‐making related to the acceptance of this article for publication. S.L.F. receives funding from the National Health and Medical Research Council, Australia, outside the submitted work. M.C.T. receives funding from NIH, Weston Brain Foundation, Tanenbaum Institute for Research in Science of Sport, Canadian Institutes of Health Research, and in‐kind funding from Roche. She has served as an advisor to Eisai, Lilly, and Novo Nordisk; she conducts clinical trials for Biogen, Anavex, Janssen, Novo Nordisk, Merck, Green Valley, and UCB; this does not reach the lower limits of funds that should be reported are specified in the JSNP's conflicts of interest policy. G.G.K. reports personal fees from Parexel, this does not reach the lower limits of funds that should be reported are specified in the JSNP's conflict of interest policy. Other funding from Rossy Family Foundation, from Edmond Safra Foundation, grants from Krembil Foundation, MSA Coalition, MJ Fox Foundation, Parkinson Canada, NIH, Canada Foundation for Innovation, and Ontario Research Fund outside the submitted work; in addition, G.G.K. has a shared patent for 5G4 Synuclein antibody and a pending patent for Diagnostic assays for movement disorders (18/537455), and received royalties from Wiley, Cambridge, and Elsevier publishers. CHT receives funding from the University Health Network Foundation, the Canadian Institutes of Health Research, and BioNTech Company.

## Data Availability

The data that support the findings of this study are available from the corresponding author upon reasonable request.
